# The Optic Canal: A Bottleneck for Cerebrospinal Fluid Dynamics in Normal-Tension Glaucoma?

**DOI:** 10.3389/fneur.2017.00047

**Published:** 2017-02-23

**Authors:** Achmed Pircher, Margherita Montali, Jatta Berberat, Luca Remonda, Hanspeter E. Killer

**Affiliations:** ^1^Department of Ophthalmology, Cantonal Hospital, Aarau, Aargau, Switzerland; ^2^Department of Neuroradiology, Cantonal Hospital, Aarau, Aargau, Switzerland

**Keywords:** glaucoma, normal-tension glaucoma, optic canal, cerebrospinal fluid, optic nerve

## Abstract

**Purpose:**

To report on the optic canal cross-sectional area (OCA) in Caucasian patients with normal-tension glaucoma (NTG) compared with Caucasian control subjects without known optic nerve (ON) diseases.

**Methods:**

Retrospective analysis of computed tomographic images of the cranium and orbits in 56 NTG patients (30 females and 26 males; 99 of 112 eyes; mean age 67.7 ± 11.1 years). Fifty-six age- and gender-matched subjects (mean age: 68.0 ± 11.2 years) without known ON diseases served as controls. The OCA at the orbital opening was measured in square millimeters by using the tool “freehand.” Statistical analysis was performed by using the independent two-tailed *t*-test.

**Results:**

The mean orbital opening OCA in NTGs measured 14.5 ± 3.5 mm^2^ (right OCA: 14.4 ± 3.6 mm^2^, left OCA: 14.5 ± 3.4 mm^2^) and in controls measured 18.3 ± 2.6 mm^2^ (right OCA: 18.5 ± 2.7 mm^2^, left OCA: 18.1 ± 2.5 mm^2^). The difference between NTG and controls was statistically significant (*p* < 0.000 for the right OCA, *p* < 0.000 for the left OCA).

**Conclusion:**

This study demonstrates narrower OCAs in Caucasian NTG patients compared with Caucasian control subjects without known ON diseases. Narrower OCAs might contribute to a discontinuity of the cerebrospinal fluid flow between the intracranial and orbital subarachnoid space in NTG patients. This might have an influence onto the pathophysiology in NTG.

## Introduction

Normal-tension glaucoma (NTG) is a multifactorial disease of the optic nerve (ON) consistent with primary open-angle glaucoma (POAG), despite a normal intraocular pressure (IOP) (1). Several IOP-independent factors such as vascular dysregulation (2), oxidative stress (3), and autoinflammatory processes (4) have been considered in the pathogenesis of NTG. The high prevalence of NTG in East Asians compared to Caucasians (5) suggests that genetic factors may play an important role. However, the pathophysiology of POAG and in particular of NTG is still poorly understood.

Recently, the role of cerebrospinal fluid (CSF) has gained interest, and CSF circulatory dysfunction has been discussed as a contributing factor (6). Several clinical and experimental studies (7–12) demonstrated a discontinuity of CSF flow along the ON.

Within the different portions of the ON, the subarachnoid space (SAS) size varies with the smallest diameter in the intracanalicular portion (13). The optic canal (OC) might therefore be a crucial location within the ON CSF pathway.

The OC lies between the body and the roots of the lesser wing of the sphenoid bone and connects the intracranial CSF spaces with the SAS of the intraorbital ON. The OC consists of bone—the static component in which the dura mater fuses with the periosteum, and the meningothelial cells (MECs)—the dynamic component which covers the pia–arachnoid layer (13). The shape of the OC varies and is described to be hour-glass, cone or cylindrical shaped (14, 15). The lumen, length, and wall thickness of the OC are highly variable along its course and shows large variation between healthy individuals and between different studies (14, 16).

This study invests the optic canal cross-sectional area (OCA) at the orbital opening with computed tomographic (CT) images in 56 Caucasian NTG patients and compared them to an age- and sex-matched control group of 56 Caucasian subjects without known ON diseases.

The study was performed to investigate about a possible anatomic predisposition in the bony part of the OC in NTG patients, which might contribute to an impaired CSF flow between the intracranial and the intraorbital SAS as demonstrated in NTG (7).

## Materials and Methods

The study was approved by the local ethical commission (Ethikkommission Nordwest- und Zentralschweiz) and follows the tenets of the Declaration of Helsinki.

The current study is a retrospective analysis of medical records of Caucasian patients with NTG and an age- and gender-matched Caucasian control group who underwent CT scan of the cranium and the orbits. The optic canal cross-sectional area (OCA) at the orbital opening in the two groups was measured and compared.

### Normal-Tension Glaucoma

Medical records of patients with POAG from 2005 to 2015 of the Department of Ophthalmology, Cantonal Hospital, Aarau, Switzerland, were analyzed. All patients who fulfilled the inclusion criteria for NTG and had undergone a CT scan of the cranium were included. In all NTG patients, CT scan was combined with cisternography that was performed if visual field defects showed progression, despite a low IOP. The informed consent from the subjects was checked before including in this study.

Normal-tension glaucoma was diagnosed on the base of glaucomatous optic morphology (optic disk cupping on ophthalmoscopy) and concomitant visual field defects. IOP maximum (untreated and treated) was always <21 mmHg, and the visual field mean deviation (MD) was ≥3 dB shown by using standard automated perimetry (SAP) (Program G2, Octopus Haag-Streit, Switzerland) at time of CT cisternography. Ophthalmoscopy, interpretation of the ON head, and SAP were performed by two experienced glaucoma specialists (Hanspeter E. Killer and Gregor Jaggi).

Each patient underwent full ophthalmologic examination including slit lamp-assisted biomicroscopy, applanation tonometry, gonioscopy, measurement of central corneal thickness, SAP, and neuroretinal rim assessment by optic coherence tomography (Heidelberg Engineering, CA, USA). In each patient, IOP was measured at least four times at different times during 24 h (between 8:00 a.m. and 8:00 p.m.) in a seated position, using Goldmann applanation tonometry and twice at night (between 9:00 p.m. and 6:00 a.m.) in a supine position, using Perkins tonometry at same day as CT cisternography was obtained. All IOP measurements were examined for its dependence on central corneal thickness in order to exclude false-negative values (17). IOP lowering treatment consisted of topical applied prostaglandin analogs, b-blockers, carbonic anhydrase, a-agonists, and combinations of these medications.

### Controls

From the database of the Department of Neuroradiology, Cantonal Hospital, Aarau, Switzerland, 56 age- and gender-matched Caucasian individuals without known intracranial or ON diseases who underwent CT of the cranium were recruited in a retrospective manner and served as controls.

All patients who underwent CT of the cranium as part of work-up for suspected cerebral stroke in the period from 2010 to 2015 were used as the initial dataset. Fifty-six age- and gender-matched patients without documented intracranial or ON diseases and without head trauma or fractions of the skull and face were included. Exclusion and inclusion criteria were verified in the medical records and by a personal conversation *via* phone in most of the controls.

For CT scan, a 64-detector scanner (Aquillion 64, Toshiba, Tokyo, Japan) providing 0.5 mm ×32 section collimation was used. Scanning parameters were a 25-cm field of view with a 512 × 512 matrix, and a soft tissue and a bone reconstruction algorithm were employed. The field of view included the foramen magnum and the nose.

Computed tomography of the head in the NTG group was combined with cisternography.

Multiplanar reconstruction images were obtained in the axial, coronal, and sagittal planes with a 0.5-mm slice thickness. CT images were analyzed using the program VitreaCore (Vital Images, Inc., Minnetonka, MN, USA) on the Advantage Workstation 4.1 software (General Electric, Milwaukee, WI, USA). All measurements were made using the same window (vertebrae window), contrast, and brightness.

### OCA Measurement

The optic canal cross-sectional area (OCA) at the orbital opening was measured in the coronal plane after the CT images have been formatted into a coronal oblique plane orthogonal to the sagittal and axial axes of each OC in order to avoid structural deformation because of oblique CT sections.

**Figure 1:**
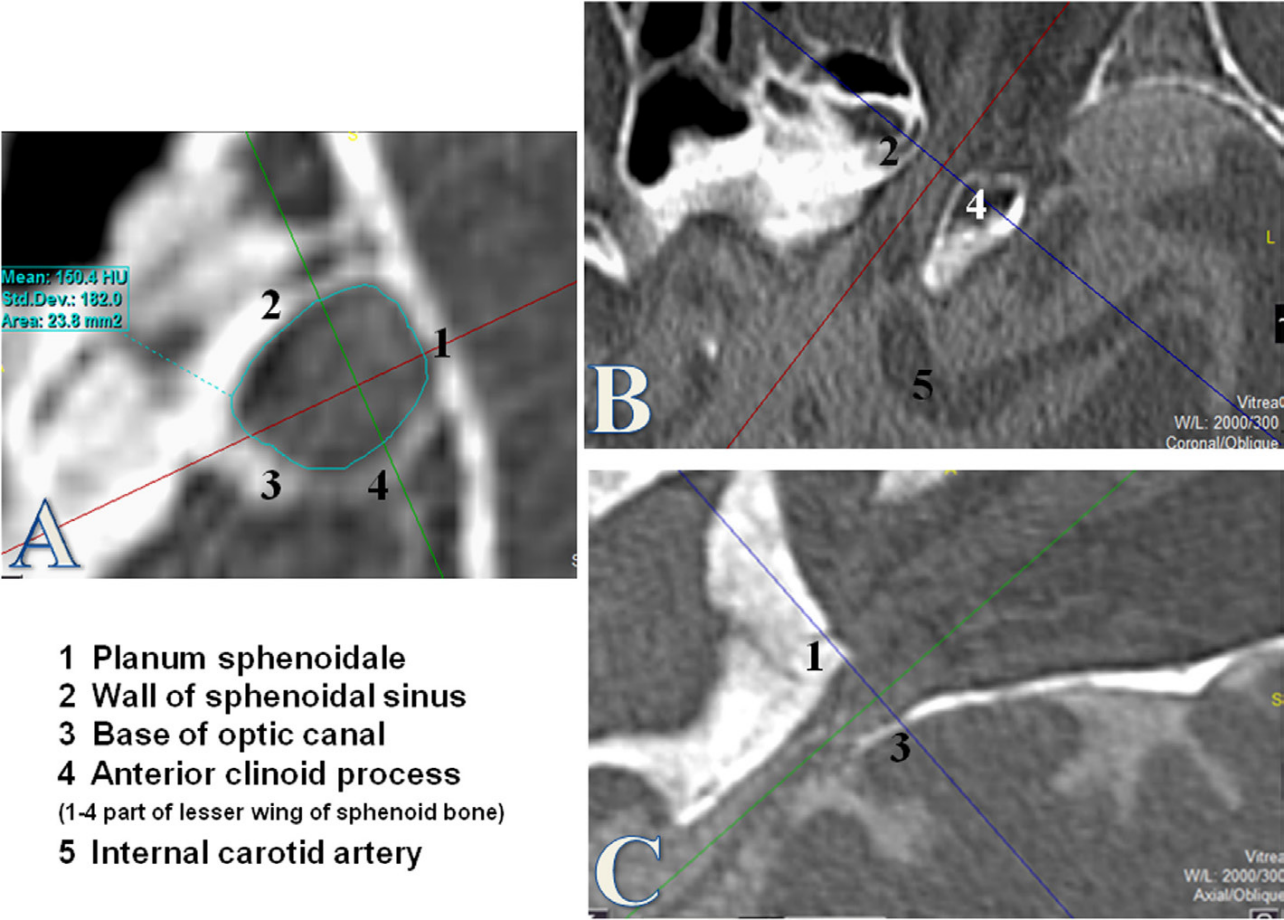
**Measurement of the optic canal cross-sectional area (OCA) at the orbital opening**. Measurements of the OCA in square millimeters by using the tool “free hand.” The orbital opening was defined as the first over 360° visible bony structure from the orbital side where the optic nerve enters through the optic canal (OC). The coronal plane **(A)** was used for measurements of the OCA after formatting into a coronal oblique plane orthogonal to the axis of the OC. **(B)** Axial plane of the OC. **(C)** Sagittal plane of the OC.

The region of interest (ROI) was defined as the first over 360° visible bone structure at the orbital side, corresponding to the orbital opening of the OC (Figure 1). The cross-sectional area was measured by using the tool “freehand” in order to consider the irregular and individual shapes of the OC.

All OCA measurements were measured twice and reviewed by an experienced neuroradiologist blinded to the neuro-ophthalmological examination.

Statistical analysis was performed using Microsoft Excel 2010 (Microsoft Corporation, Redmond, WA, USA), and the SPSS 21.0 (IBM SPSS Inc., Chicago, IL, USA) for Windows statistical package. The two-tailed *t*-test for unpaired samples was used for analysis of the differences between NTG subjects and controls, right and left ON, and between males and females. The non-parametric Spearman rank order correlation coefficient was used for analysis of correlations between OCA and age.

## Results

Fifty-six *patients* (mean age 67.7 ± 11.1 years), 30 women (68.1 ± 10.7 years) and 26 men (67.2 ± 11.8 years): 99 of 112 eyes (53 eyes in females and 46 eyes in males), fulfilled the inclusion criteria for NTG (Table 1). The mean glaucomatous visual field defect (MD) at time of CT was 13.9 ± 7.1 dB on the right eye and 13.5 ± 7.3 dB on the left eye. The mean IOP was 14.1 ± 2.6 mmHg on the right eye and 14.1 ± 2.5 mmHg on the left eye.

**Table 1:** **Measurement of the optic canal cross-sectional area (OCA) at the orbital opening**.

	NTG patients		OCA (mm^2^)		Controls		OCA (mm^2^)	
*N*	Age f	Age m	Right	Left	Age f	Age m	Right	Left
1	45		–	14.9	44		–	15.5
2		45	13.7	12.9		48	16.7	17.1
3		50	20.7	19.0		52	18.7	18.2
4		51	18.4	21.0		53	21	19.0
5	52		–	17.1	51		–	18.9
6		52	18.1	17.9		54	19.7	18.7
7	53		8.9	8.3	53		17.8	17.1
8		53	12.5	12.0		58	24.8	20.5
9		55	17.6	17.6		58	22.9	19.8
10	55		15.6	14.5	53		15.2	15.5
11	57		10.8	11.4	53		18.1	18.4
12	58		10.9	14.1	55		15.6	13.9
13	58		7.7	8.2	57		18.7	20
14		59	13.3	–		58	16.3	–
15	60		10.6	11.9	60		20.6	19.2
16		61	19.9	18.5		61	20.2	19.8
17		61	–	14.0		61	–	19.5
18		63	–	13.1		65	–	15.0
19		63	–	16.8		65	–	15.7
20	64		15.8	14.2	61		19.7	20.4
21	64		15.6	13.0	62		20.8	16.7
22	64		19.3	22.4	62		17	19.8
23		64	14.1	15.9		65	19.7	19.5
24	65		11.4	14.5	65		15.4	16.2
25	65		12.6	14.2	66		21.8	22.6
26		65	14.5	11.4		67	16.2	12.8
27	66		10.9	14.1	66		16.8	15.7
28	67		–	10.6	67		–	20
29	68		14.9	–	68		18.5	–
30		69	15.2	15.2		67	21.0	19.5
31	69		15.7	–	69		16.9	–
32		71	11.3	10.9		73	20.2	21.3
33	72		13.0	12.0	71		17.4	13.1
34	72		13.6	12.3	72		17.7	19.5
35		73	22.0	–		74	16.7	–
36	73		17.6	14.7	73		17.8	20.5
37		73	13.3	12.1		75	15.7	17.3
38		74	12.3	19.8		77	19.7	18.6
39	74		17.4	14.1	73		16.8	17
40	75		–	20.3	76		–	20.2
41	75		12.4	14.0	78		20.2	18.1
42	76		13.0	14.2	79		19.8	19.6
43		76	13.6	12.0		77	13.6	15.3
44		77	10.5	10.8		78	22.7	20.3
45	77		11.2	11.2	79		20.2	22.1
46		78	–	11.7		78	–	16.3
47		79	12.0	13.0		79	23.5	22.1
48	79		12.3	12.9	80		16.3	17.2
49	80		9.3	9.6	81		16	16.5
50		80	18.5	18.5		81	17.4	16.5
51		81	20.0	21.8		82	19.7	17.5
52	83		13.2	12.6	83		23	21.5
53		85	23.2	19.2		83	19.0	21.4
54	86		–	15.2	83		–	17.6
55		88	17.7	17.9		88	11.4	12.3
56	91		11.9	13.1	92		15.5	14.9
Mean ± SD	68.1 ± 10.7	67.2 ± 11.8	14.4 ± 3.6	14.5 ± 3.4	67.7 ± 11.5	68.3 ± 11.1	18.5 ± 2.7	18.1 ± 2.5

*Measurement of the optic canal cross-sectional area (OCA) in square millimeters at the orbital opening defined as the first over 360° visible bony structure from the orbital side where the optic nerve (ON) enters through the OC. Age for male (m) and female (f) in years*.

*NTG, normal-tension glaucoma; OCA, optic canal cross-sectional area; SD, standard deviation*.

The *control group* consists of 56 age- and gender-matched controls without known ON diseases: corresponding to the NTG group 99 of 112 eyes (53 eyes in females and 46 eyes in males) were included. Mean age was 68.0 ± 11.2, 67.7 ± 11.5 in females, and 68.3 ± 11.1 in males (Table 1).

Subjects in the NTG and control groups showed no statistically significant difference in either gender or age (*p* = 0.866).

### Optic Canal

The mean OCA at the orbital opening in all *NTG patients* in both eyes (*n* = 99) was 14.5 ± 3.5 mm^2^. On the right side (*n* = 47), mean OCA was 14.4 ± 3.6 mm^2^; in females (*n* = 25), it ranged from 7.7 to 19.3 mm^2^ (mean: 13.0 ± 2.9) and in males (*n* = 22) from 10.5 to 23.2 mm^2^ (mean: 16.0 ± 3.7). Mean OCA on the left side (*n* = 52) was 14.5 ± 3.4 mm^2^ and ranged from 8.2 to 22.4 mm^2^ (mean: 13.6 ± 3.0) in females (*n* = 28) and from 10.8 to 21.8 mm^2^ (mean: 15.5 ± 3.5) in males (*n* = 24) (Table 1). Between females and males, a statistical significant difference was shown for the right OCA (*p* = 0.004) and for the left OCA (*p* = 0.035). No statistical significant difference was between right and left OCA neither in females (*p* = 0.513) nor in males (*p* = 0.657).

In the *control group*, the mean orbital opening OCA in both eyes (*n* = 99) was 18.3 ± 2.6 mm^2^. Mean OCA on the right (*n* = 47) was 18.5 ± 2.7 mm^2^; in females (*n* = 25), it ranged from 15.2 to 23.0 mm^2^ (mean: 18.1 ± 2.1) and in males (*n* = 22) from 11.4 to 24.8 mm^2^ (mean: 19.0 ± 3.3). Mean OCA on the left (*n* = 52) was 18.1 ± 2.5 mm^2^ and ranged from 13.1 to 22.6 mm^2^ (mean: 18.1 ± 2.5) in females (*n* = 28) and from 12.3 to 22.1 mm^2^ (mean: 18.1 ± 2.6) in males (*n* = 24) (Table 1). Neither between females and males (right OCA: *p* = 0.331; left OCA: *p* = 0.945) nor between right and left OCA, a statistical significant difference was shown (*p* = 0.438).

**Figure 2:**
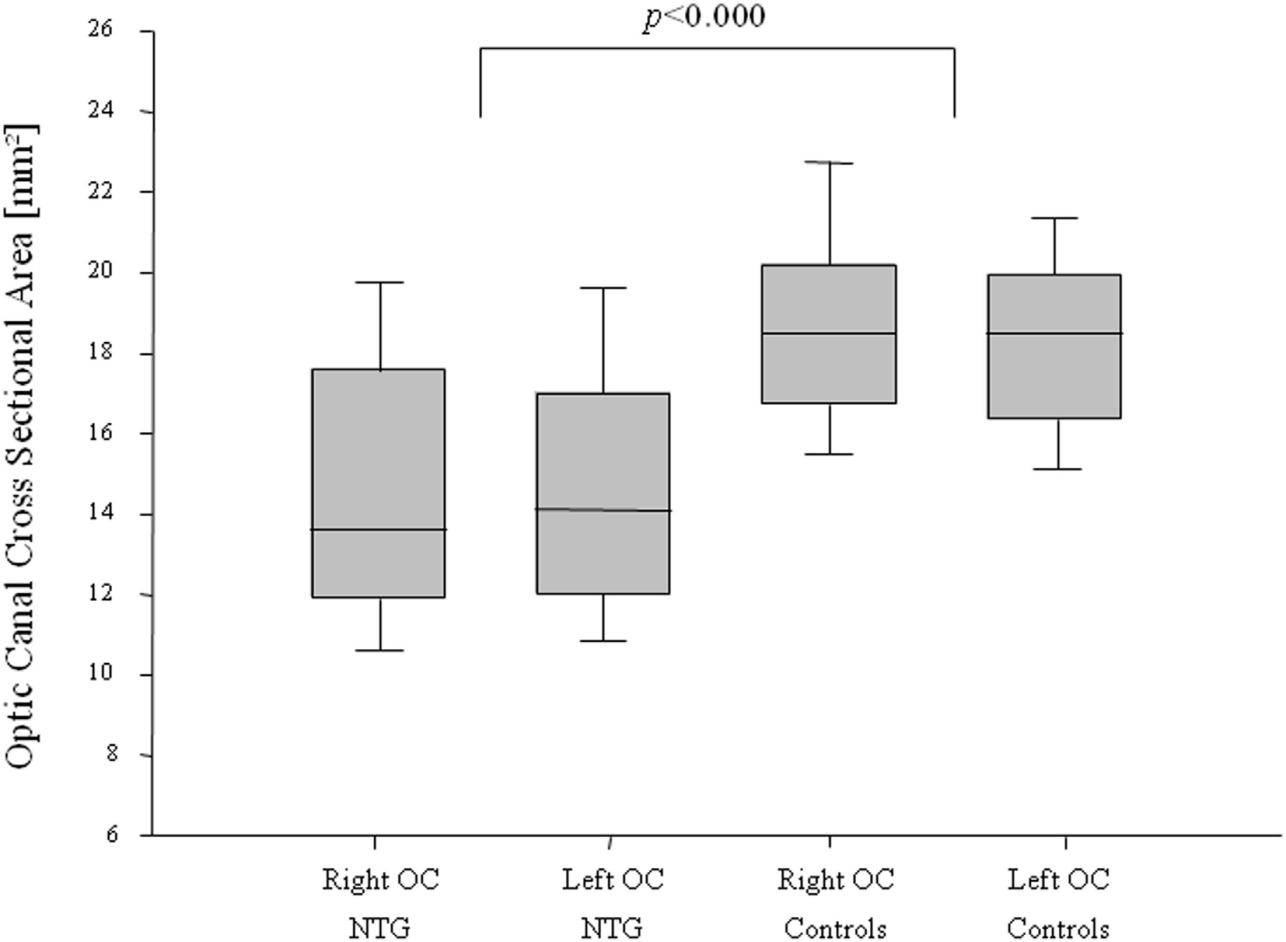
**Optic canal cross-sectional area (OCA) in normal-tension glaucoma (NTG) patients and controls**. Box plot of optic canal cross-sectional area (OCA) measurements in NTG patients and controls, separated for right and left OCA. NTG patients (*n* = 56; right OCA: *n* = 47, left OCA: *n* = 52) and controls (*n* = 56, right OCA: *n* = 47, left OCA: *n* = 52).

A statistically significant difference in the orbital opening OCA was found between NTG patients and controls for the right and for the left side (*t*-test for right OCA: *p* < 0.000, left OCA: *p* < 0.000) (Figure 2). The difference showed statistical significance in females and males for the right OCA (females: *p* < 0.000; males: *p* = 0.008) and for the left OCA (females: *p* < 0.000, males: *p* = 0.007).

Bivariate analysis comparing OCA with age neither showed statistically significant correlation nor was there a correlation for the right nor for the left side neither in the NTG group (Spearman’s correlation coefficient for the right OCA, ρ = −0.03, *p* = 0.833; for the left OCA, ρ = −0.06, *p* = 0.693) nor in the control group (Spearman’s correlation coefficient for the right OCA, ρ = −0.06, *p* = 0.709; for the left OCA, ρ = 0.05, *p* = 0.739).

## Discussion

This study demonstrates a significantly smaller optic canal cross-sectional area (OCA) at the orbital opening in 56 Caucasian NTG patients compared with 56 age- and gender-matched Caucasian control subjects without ON diseases.

The role of the OC and its impact on CSF dynamics have recently been investigated in clinical and experimental studies (9–12). Bidot et al. demonstrated in a retrospective analysis with magnetic resonance imaging (MRI) an association between the OC size and the severity of papilledema in patients with idiopathic intracranial pressure (IIH) (11). Severe papilledema and poor visual function were associated with a larger OCA in 69 patients with IIH (11). Accordingly, a smaller OCA was associated with the side of less sever papilledema in eight patients with asymmetric papilledema (10). These findings suggest that the OC substantially influences the CSF inflow into the orbital SAS.

In a prospective cohort study, Bekerman et al. measured the narrowest OCA in CT scans in 54 patients with intracranial pressure (ICP) monitoring and 600 controls (12) and concluded that in cases with narrow OCs, the ON sheath diameter (ONSD) monitoring provides false-negative results. The authors explained their findings with a possible reduced flow from intracranial to intraorbital and therefore a lower pressure in the SAS of the ON (12).

Further evidence was presented by Hou et al. (9) who performed an experimental model in eight dogs. Hou et al. demonstrated if the ICP (measured in the left brain ventricle) falls below a significant certain level (“breakpoint”), the correlation between ICP and optic nerve subarachnoid space pressure (measured in the retrobulbar ON SAS) was getting lost. The authors postulated an obstruction of the CSF inflow through the OC as one possible explanation for their findings (9).

An important role for CSF dynamics within the ON SAS is played by MECs. MECs are multifunctional cells covering the meninges in the entire central nervous system. MECs cover the pia and the arachnoid layer of the meninges as well as the septae and trabeculae in the SAS of the brain and the ON (13). They were shown to provide a barrier between CSF and neuronal tissue and between CSF and blood stream (18, 19). MECs react with proliferation and growth to mechanical stimuli such as pressure, while pressure on the other hand reduces the phagocytic activity of MECs (20). Fluctuations of ICP over time are normal and frequent. Increased ICP, e.g., during Valsalva maneuver (that can triple the CSF pressure) can be a factor that induces proliferation and growth of MECs, thereby leading to a narrowing of the SAS diameter causing CSF stasis. Such MEC proliferation has been demonstrated to be more abundant in patients with POAG compared with healthy subjects (21).

Within the OC, the SAS measures the smallest diameter (13). Here, the dura mater fuses with the periosteum. Due to its bony confinement, there is no possibility for the SAS to expand, and a proliferation of MECs will lead inevitable to a narrowing of this anyway narrow portion with its bottleneck characteristics.

There are a few studies in the literature, which report on OCA measurements. The data show large variations between healthy individuals and between different studies (12, 14, 16, 22). Bekerman et al. (12) measured in 600 control subjects on CT images the diameter of the OCA and ONSD in order to evaluate the influence of the bony canal for ONSD monitoring. In this work, Bekerman et al. (12) calculated the cross-sectional area as a function of the diameter. The calculated orbital opening OCA in their study was 15.8 mm^2^ on the right side and 16.5 mm^2^ on the left side. These measurements show a smaller orbital opening OCA compared with our control subjects. However, unlike our study, the areas were calculated as a function of the diameter, which might be a difference as the shape of the canal is irregular. Similar to our results, their measurements showed large variations within individuals.

An even larger cross-sectional area compared to our measurements of the control group was measured in a population from China on spiral CT images by Jiang et al. (22). The calculated cross-sectional area related to their diameter measurements would be 23.8 mm^2^ on the right side and 24.4 mm^2^ on the left side.

Such differences are likely the result of different measuring methods (diameter vs. free hand tool) and imaging techniques (CT vs. MRI), and the individual anatomy between different races (Asian vs. Caucasian, etc.) and measurement inaccuracies due to the irregular and individual variable shapes. As the OC volume declines over time (23), age as well may have an influence on the outcome of the measurements. A comparison with other studies might therefore not render conclusive results.

However, the measurement method and technique in our study were the same in the NTG group and control subjects (age matched) as well. Right and left OCs showed similar data in both NTGs (OD = 14.4 ± 3.6 mm^2^, OS = 14.5 ± 3.4 mm^2^) and controls (OD = 18.5 ± 2.7 mm^2^, OS = 18.1 ± 2.5 mm^2^).

An interesting finding in this study was a difference between females and males. This difference was statistically significant only in the NTG group (right OCA: *p* = 0.004, left OCA: *p* = 0.035). Whether this association plays a role for the slightly more rapid progression of the disease in untreated female NTG patients compared with males (24) is speculative. However, smaller OCs in females were also reported in other studies (23).

A limitation of our study is that we could not measure the SAS annulus surrounding the ON within the OC. CT imaging does not allow us to distinguish the exact borders between the ON and ON sheath from the CSF-filled SAS. We used in our study the cross-sectional area at the orbital opening (first over 360 grad visible bony structure) as ROI because the orbital opening is much easier to standardize on our CT images. This provides a more accurate comparison between different individuals. We are aware that for a proper evaluation of the fluid dynamic impact, it would be necessary to measure the entire volume of the SAS within the individually and irregularly shaped OC.

In this study, the OCA at the orbital opening in the NTG group measured a significantly (*p* < 0.000) smaller area compared to age- and gender-matched controls without ON diseases. This anatomical setting might act as a bottleneck and could predispose to partial or complete obstruction of the SAS within the canalicular portion as seen in patients with papilledema (11, 25) as well as in patients with NTG (7). Its possible role in the pathophysiology of NTG *via* a change in CSF dynamics and content needs to be further evaluated.

## Author Contributions

All the authors listed have contributed significantly and are in agreement with the content of the manuscript. AP was mainly involved in study design, data analysis, data interpretation, and manuscript preparation. MM was mainly involved in study design, data acquisition, data interpretation, and manuscript preparation. AP and MM contributed equally to this work. JB and LR were mainly involved in data acquisition and data analysis. HK was mainly involved in study design, data interpretation, and manuscript preparation.

## Conflict of Interest Statement

The authors declare that the research was conducted in the absence of any commercial or financial relationships that could be construed as a potential conflict of interest.

## Abbreviations

OCA, optic canal cross-sectional area; NTG, normal-tension glaucoma; ON, optic nerve; CT, computed tomographic; CSF, cerebrospinal fluid; SAS, subarachnoid space; POAG, primary open-angle glaucoma; IOP, intraocular pressure; MECs, meningothelial cells; OAG, open-angle glaucoma; MD, mean deviation; SAP, standard automated perimetry; OCT, optic coherence tomography; ROI, region of interest; IIH, idiopathic intracranial pressure; ONSD, optic nerve sheath diameter; ICP, intracranial pressure; MRI, magnetic resonance imaging.
